# Supplementary material for: Causal Relationship between Aging and Anorexia Nervosa: A White-Matter-Microstructure-Mediated Mendelian Randomization Analysis
Source: Biomedicines. 2024 Aug 16;12(8):1874. doi: 10.3390/biomedicines12081874 (PMC11351342; doi:10.3390/biomedicines12081874)
Supplement: Supplementary file 1 [file biomedicines-12-01874-s001.zip › Supplementary Figure/Figure S4/Singe SNP plot of MR analysis for TL to ubm-b-1600.pdf]

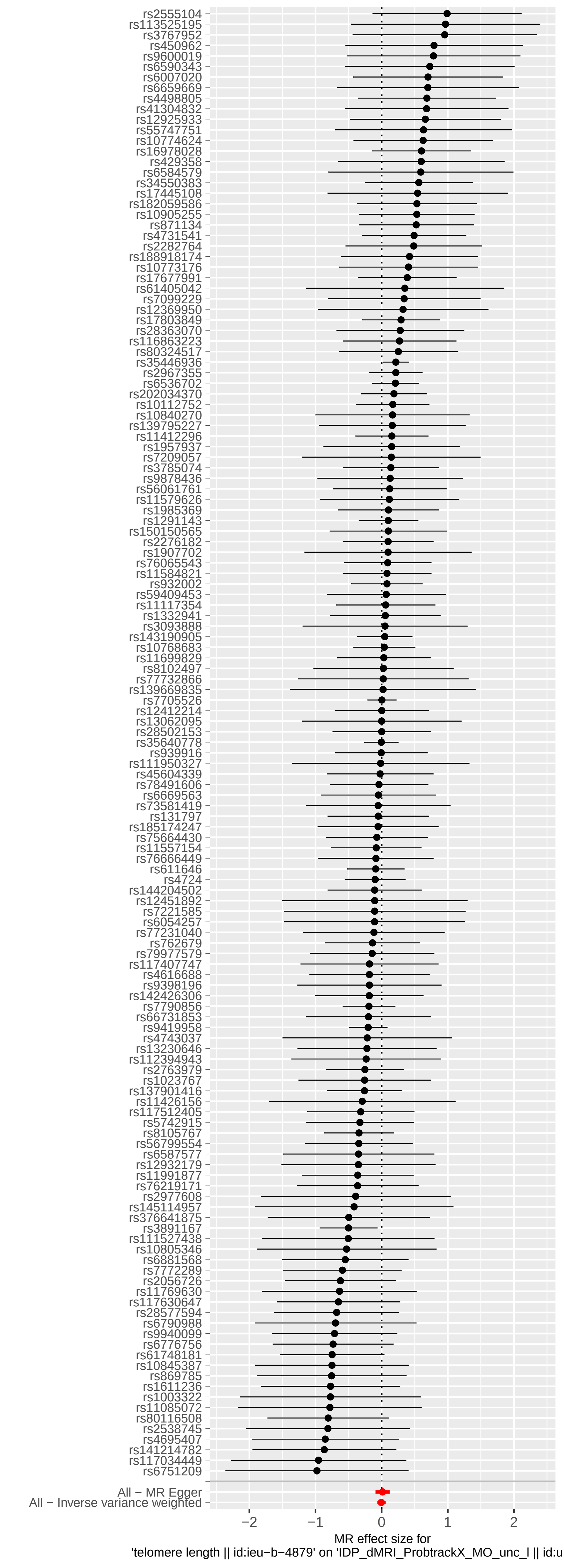

rs2555104
rs113525195
rs3767952
rs450962
rs9600019
rs6590343
rs6007020
rs6659669
rs4498805
rs41304832
rs12925933
rs55747751
rs10774624
rs16978028
rs429358
rs6584579
rs34550383
rs17445108
rs182059586
rs10905255
rs871134
rs4731541
rs2282764
rs188918174
rs10773176
rs17677991
rs61405042
rs7099229
rs12369950
rs17803849
rs28363070
rs116863223
rs80324517
rs35446936
rs2967355
rs6536702
rs202034370
rs10112752
rs10840270
rs139795227
rs11412296
rs1957937
rs7209057
rs3785074
rs9878436
rs56061761
rs11579626
rs1985369
rs1291143
rs150150565
rs2276182
rs1907702
rs76065543
rs11584821
rs932002
rs59409453
rs11117354
rs1332941
rs3093888
rs143190905
rs10768683
rs11699829
rs8102497
rs77732866
rs139669835
rs7705526
rs12412214
rs13062095
rs28502153
rs35640778
rs939916
rs111950327
rs45604339
rs78491606
rs6669563
rs73581419
rs131797
rs185174247
rs75664430
rs11557154
rs76666449
rs611646
rs4724
rs144204502
rs12451892
rs7221585
rs6054257
rs77231040
rs762679
rs79977579
rs117407747
rs4616688
rs9398196
rs142426306
rs7790856
rs66731853
rs9419958
rs4743037
rs13230646
rs112394943
rs2763979
rs1023767
rs137901416
rs11426156
rs117512405
rs5742915
rs8105767
rs56799554
rs6587577
rs12932179
rs11991877
rs76219171
rs2977608
rs145114957
rs376641875
rs3891167
rs111527438
rs10805346
rs6881568
rs7772289
rs2056726
rs11769630
rs117630647
rs28577594
rs6790988
rs9940099
rs6776756
rs61748181
rs10845387
rs869785
rs1611236
rs1003322
rs11085072
rs80116508
rs2538745
rs4695407
rs141214782
rs117034449
rs6751209
All − MR Egger
All − Inverse variance weighted
−2
−1
0
1
2
MR effect size for
'telomere length || id:ieu−b−4879' on 'IDP_dMRI_ProbtrackX_MO_unc_l || id:u
